# Supplementary material for: Are carbon emissions trading and green financial instruments synergistic? -Comprehensive quantitative research based on content analysis
Source: PLoS One. 2024 Mar 7;19(3):e0298601. doi: 10.1371/journal.pone.0298601 (PMC10919601; doi:10.1371/journal.pone.0298601)
Supplement: S1 Data — (ZIP) [file pone.0298601.s001.zip › Data/地级市Z_F工作报告.zip!/║╙─╧/┬σ╤⌠╩╨/└┤╫╘╣1⁄2╓┌║┼í╛┬φ┐╦╩2╛▌═°í┐_2008.docx]

各位代表：

　　现在，我代表市人民政府向大会作工作报告，请予审议，并请各位政协委员和列席人士提出意见。

　　一、2007年工作回顾

　　2007年，在省委、省政府和市委的正确领导下，在市人大、市政协的监督支持下，市人民政府团结带领全市人民，以科学发展观为指导，抢抓机遇，加快发展，较好地完成了市十二届人大四次会议确定的目标任务。

　　——国民经济快速增长，综合实力再上新台阶。预计全年生产总值1570亿元左右，同比增长15%以上；其中一、二、三产业分别增长5%、18.8%和12%。财政一般预算收入98亿元、增长28%，支出135亿元、增长20.2%。规模以上工业增加值610亿元，增长25%。全社会固定资产投资780亿元、增长25%，其中工业投资410亿元、增长35%。社会消费品零售总额466亿元，增长18%。进出口总额13.4亿美元、增长28.8%，其中出口10.2亿美元、增长30.7%。城镇居民人均可支配收入和农民人均纯收入实际增长10%和11%。11月底，全市金融机构存贷款余额分别达到1152.4亿元和620.1亿元，比年初增加93.5亿元和72.2亿元。

　　——项目建设加快推进，招商引资成效显著。强力实施项目带动。建立项目例会、项目周报、市领导分包项目制度，狠抓项目谋划、协调和落实，预计全年实施各类项目2900个，总投资1500亿元以上，完成投资700亿元左右。其中，千万元以上工业结构调整项目400个，完成投资230亿元。中硅公司1000吨多晶硅、香江万基40万吨氧化铝、尚德公司90兆瓦太阳能电池及组件、一拖集团大中马力轮式拖拉机等196个项目竣工投产。香江万基氧化铝二期、伊电集团50万吨连铸连轧、洛钼集团4万吨钼冶炼、阿特斯3000吨长晶及硅片、洛玻140万平方米低辐射镀膜玻璃、昇阳硅业20万吨工业硅等240个项目开工建设。洛阳石化1000万吨炼油扩能、45万吨PX、20万吨聚丙烯、100万吨二甲醚、华阳孟津电厂、渠里煤矿改扩建等重大项目前期取得实质性进展，获国家、省、市审批、核准、备案项目890个。华阳石化后加工、金堆城钼业电子玻璃、埃索菲通公司万吨多晶硅、伊电集团2×600兆瓦机组、新电集团2×300兆瓦机组和40万吨铝板带箔、永安特钢搬迁改造等一批新策划项目取得明显进展。招商引资力度进一步加大。建立完善招商引资奖励机制，中博会、牡丹花会和豫商大会等重大招商活动取得圆满成功。预计全年利用市外境内资金150亿元，增长34.4%。新批外商投资企业43家；实际利用外资6.37亿美元，增长2倍。全年争取政策性资金有望突破6亿元。

　　——工业结构不断优化，经济效益大幅提升。六大优势产业日益壮大。预计规模以上工业主营业务收入2100亿元、增长35%；利税265亿元、增长1倍以上，利润170亿元、增长1.4倍，增速均居全省第一。预计全年六大优势产业占规模以上工业增加值的86%。新兴产业培育取得重大进展。出台了一系列促进轴承、五车(机)零部件、纺织服装等高成长性关联产业、特色产业和高新技术产业发展的政策措施。总投资22.9亿元的41个五车(机)零部件项目已经备案。一批新策划的机械制造高端项目、精细化工项目、医疗设备项目正在开展前期工作。重点企业实力明显增强。新电集团、伊电集团、一拖集团跻身中国企业500强。一拖集团等4家企业6个产品获中国名牌称号。预计主营业务收入超亿元企业200家，其中超50亿元企业7家、超百亿元企业4家、超300亿元企业1家。科技创新能力不断提高。获国家科技进步奖5项，省科技进步一等奖2项、二等奖15项，其中超薄浮法玻璃关键技术获国家科技进步一等奖，是改革开放以来我省工业领域首个获奖项目。新建省级工程技术中心和重点实验室8个，市级研发机构52个。争取国家“863”计划项目5个，国家、省科技项目51个。高技术产业增加值增长1.1倍。高新技术产业开发区预计完成技工贸总收入485亿元，实现利税36亿元。

　　——新农村建设扎实推进，农村经济健康发展。财政支农力度持续加大，落实粮食直补和各类专项补贴2.84亿元。新农村建设成效显著。完成了666个村建设规划和757个村控制性规划。“村村整治、千村达标、百村示范”工程加快推进，村容村貌、路容路貌明显改观。农村沼气、安全饮水等公共设施建设进展顺利。主导产业不断壮大。预计粮食总产236万吨、水果45万吨、肉蛋奶总产80万吨，均创历史新高。烟叶面积、收购量和税收居全省第一。我市被确定为全省唯一的国家循环农业示范市。发展龙头企业52家，新增省级龙头企业7家，农村专业合作组织109家，带动农户65万户以上，产业化收入占农民总收入的48%。农村基础设施不断完善。孟西灌区即将竣工，前坪水库项目通过省级初审。治理水土流失205平方公里，新增有效灌溉面积3.5万亩，改造中低产田10.6万亩。农村困难群众危房改造完成10%的建设任务。

　　——旅游业发展态势喜人，服务业水平进一步提升。旅游强市战略加快实施。洛阳旅游被纳入《丝绸之路区域旅游规划》和《中部六省区域旅游规划》。成功举办第25届牡丹花会和第4届河洛文化旅游节。伏牛山生态旅游景区建设、白马寺景区规划和印度风格佛殿工程加快推进。积极参与隋唐大运河、丝绸之路申报世界文化遗产工作，龙门石窟入选全国首批5A级旅游景区和最受群众喜爱的十大风景名胜区，龙潭峡、神灵寨、木札岭、天池山入选4A级旅游景区，栾川县跻身首批17个中国旅游强县。预计全市全年接待游客4002.5万人次、增长42.8%，其中入境游26.2万人次、增长21.1%；景点门票收入5.1亿元、增长32.9%，旅游总收入194.7亿元、增长42.4%。服务业稳步发展。预计城市规划区内完成投资12亿元，新增商业面积30万平方米。中央商务广场等建成投用，沃尔玛即将开业，宝龙城市广场等10个项目开工建设。新区新增商户335家，营业面积已达4万平方米。实施“万村千乡”市场工程，建成农家店1060家。房地产业健康发展。预计完成投资70亿元。商品房开工980万平方米、竣工280万平方米，经济适用房开工45万平方米、竣工27万平方米。房地产交易额80亿元。归集住房公积金10.7亿元，发放公积金贷款5.8亿元。

　　——节能减排深入推进，生态建设成效明显。大力实施碧水蓝天青山绿地工程。完成环评审批项目529个、环保验收72个、区域环评2个。中信重机窑炉改造等7个节能项目获国家奖励资金2966万元。关闭取缔重污染企业6家，限期或停产治理27家。关停小火电34万千瓦，对省定25家落后生产能力企业实行了差别电价。县(市)污水处理厂和垃圾处理场全部建成投用。资源集约利用成效明显。扎实开展矿产资源集中整治，关闭非法矿山、矿点366个，小煤矿由215家整合为64家。新建标准化厂房95万平方米；完成土地“三项整治”3万亩；拆除粘土砖瓦窑厂611个，在全省率先完成任务。生态建设加快推进。完成生态洛阳建设规划，积极推进北邙绿化、城郊绿化、退耕还林、天然林保护等重点生态工程建设，荒山造林17.8万亩，植树造林21.6万亩。隋唐城遗址公园二期、洛浦公园东延伸工程基本完工，新增城市绿地90万平方米。全年削减化学需氧量1.4万吨、二氧化硫10万吨，市区环境空气质量二级以上天数超过300天。

　　——改革开放步伐加快，发展活力明显增强。国有企业战略重组顺利推进。洛玻与中建材完成股权划转和变更登记，洛铜与中铝重组后新建10万吨高精度铜板带项目进展顺利，凌宇与中集重组后搬迁改造项目进入施工高潮，安龙钢铁与永通特钢成立的永安特钢生产经营良好，一拖集团与国机集团战略重组加快推进，黄河水泥等战略重组取得新进展，洛轴政策性破产近期有望获国家批准。企业上市步伐加快。洛钼集团在香港上市募集资金81亿港元，中航光电A股上市募集资金4.8亿元，一拖股份在香港完成配股、募集资金2.3亿港元。其它领域改革全面推进。机关事业单位工资制度改革和规范津补贴工作进展顺利。行政管理体制和事业单位机构改革不断深入。部门预算改革、国库收付体系和政府采购制度日趋完善。农村公路养护、城市环卫等体制改革稳步推进。县域经济实力增强。预计县(市、区)实现生产总值1330亿元左右，增长17%，占全市的85%。城镇固定资产投资356亿元，增长60%，占全市的52%；其中工业投资246亿元，增长60%。财政一般预算收入65亿元，增长31%，占全市的66%。非公有制经济快速发展。预计实现增加值680亿元、税收80亿元，分别增长38%和35%。营业收入超亿元民营企业有望突破100家，其中超5亿元企业16家、超10亿元企业4家。

　　——城市建设快速推进，城市形象大幅提升。城市规划修编进展顺利。第四期城市总体规划和土地利用总体规划修编通过省级评审。洛阳市近期建设规划、南站地区概念规划、洛河两岸景观规划及城市设计等基本完成。新区建设步伐加快。预计全年开工项目180个，完成投资80亿元。滨河百栋高层累计开工111栋175万平方米；开元大道东西延长线、定鼎门街等6条道路竣工通车，豫能阳光热电联产、定鼎门污水处理厂等32项公共设施建成投用，瀛洲桥、关林路西延长线等项目进展顺利，新区博物馆、新区医院等开工建设。洛北城区改造稳步推进。预计完成投资60亿元，武汉路、石油路等13条道路竣工通车，衡山路、七一路雨水泵站建设进展顺利。155个地块改造项目完成投资48亿元。交通基础设施建设明显加快。预计完成投资16亿元。洛阳至南阳高速汝阳段路基工程基本完工，旧邓线白河至栗树街、大青沟至内乡交界等公路建成通车，改造县乡公路107公里、通村联网水泥路1178公里。偃师至洛宁、洛阳至吉利快速通道获省核准。精细化管理水平不断提高。完善市容巡查机制，深入开展“五城联创”，荣膺国家卫生城市称号。加快社区建设，住宅小区物业管理水平不断提高。我市成功入选“倾国倾城·最值得向世界介绍的中国名城”、“感动世界的中国品牌城市”和“世界特色魅力城市200强”。

　　——社会事业全面发展，和谐社会建设不断加强。20件实事按期完成。建成农村户用沼气20.3万座、是去年的3.4倍，成立乡级服务站117家、村级网点687个；硬化通组通户道路3292公里，解决农村饮水安全24.5万人，搬迁扶贫1.2万人；新建、改扩建农村敬老院70所，五保户集中供养率30.7%；新增城乡低保对象7.8万人，累计为30.9万名低保对象发放低保金1.63亿元；建成城市社区卫生服务中心44个、服务站70个，改造农村标准化卫生所409家；新型农村合作医疗制度实现全覆盖，参合农民428万人、参合率90.5%；“两免一补”资助贫困学生248.3万人次1.9亿元；培训农村劳动力25.5万人次，转移131万人次；建成农村社区服务中心601个、乡级便民服务中心52个。改造城中村道路和背街小巷172条；棚户区改造全面启动，1365户城市困难群众享受廉租住房补贴；新增城镇就业15万人、再就业4.9万人，安置零就业家庭就业4174人，在全省率先完成任务；新增城镇基本养老保险5.97万人，基本医疗保险参保职工总数73.9万人。城市中小学D级危房改造全面完成，全部免除低保家庭子女义务教育阶段学杂费，接收进城务工人员子女义务教育学校达77所。社会保障体系日臻完善。预计征集社会保险费22.1亿元、发放25亿元，退休金标准提高到人均835元。企业养老保险费率再降1个百分点，为企业减负2.5亿元。我市被列为全国首批城镇居民基本医疗保险试点城市，已参保42万人、缴费40万人，基本形成了覆盖城乡的医疗保障体系。实施食品药品放心工程，成为全国餐饮业卫生监督公示制试点城市。社会事业不断进步。“两基”迎国检工作扎实推进，基础教育水平不断提高，普通高招创历史新高。洛阳工业高专和洛阳大学合并升本，组建了洛阳理工学院并顺利挂牌。隋唐城定鼎门遗址、宫城核心区和汉魏故城阊阖门遗址保护展示工程顺利实施。各大公园、博物馆免费开放。“河洛欢歌”、“欢乐新农村”等群众文化活动蓬勃开展。低生育水平持续稳定，人口出生率控制在11.9‰以内。我市在全国第七届残运会和世界特奥会上取得好成绩。统计、审计、外事、侨务、人防、地震、气象、地方志、民族宗教、新闻出版、发展研究等都取得了新成绩。

　　——精神文明和民主法制建设进一步加强，平安洛阳建设扎实推进。坚持依法行政，自觉接受人大及其常委会监督，支持政协参政议政，认真办理人大代表建议和政协委员提案，办结率100%、满意率99%以上。“五五”普法顺利实施。提请市人大常委会通过地方性法规4件，颁布行政规章10件。高度重视信访稳定，建立完善了连线政府、市长信箱、市长热线、市长短信、群众反映突出问题办理周报等制度，畅通群众诉求渠道，解决了一大批信访积案。安全生产形势持续好转。强化农村和社区警务建设，大力开展打黑除恶和命案攻坚专项行动，社会大局保持稳定。反腐倡廉取得新成效。社会主义荣辱观教育和精神文明创建活动不断深入。双拥工作成效显著，有望荣获全国双拥模范城五连冠。

　　各位代表，2007年是国家宏观调控进一步趋紧、发展压力明显增大的一年，也是我们加快发展、科学发展的一年。一年来，我们坚持在发展中顺应调控，在调控中谋求发展，理清工作思路，强化工作措施，发展能力进一步提高。我们坚持工业强市、旅游强市、科教强市和项目引资双带动战略，着力创新发展理念、破解发展难题，发展速度进一步加快。我们坚持推进结构调整，加快转变发展方式，狠抓节能减排和资源节约利用，发展质量进一步提高。我们坚持加强效能建设和信用体系建设，深入开展“讲正气、树新风、建绩效”活动，严查“四乱”行为，发展环境进一步优化。我们坚持统筹城乡发展和经济社会发展，着力实现好、维护好、发展好人民群众的根本利益，和谐社会建设进一步加强。

　　这些成绩的取得，是省委、省政府和市委正确领导的结果，是市人大、市政协积极支持的结果，是全市人民共同奋斗的结果。我代表市人民政府，向全市广大工人、农民、知识分子、干部，向人大代表和政协委员，向驻洛部队、武警官兵和公安干警，向各民主党派、人民团体和离退休老同志，向所有关心支持洛阳发展的社会各界，表示衷心的感谢并致以崇高的敬意！

　　肯定成绩的同时，还要看到经济社会发展和政府工作中仍存在不少矛盾和问题。经济结构不尽合理。资源性、初加工工业占规模以上工业增加值的70%以上，高技术产业仅占2.41%；农业基础相对薄弱，第三产业比重低且逐年下降；服务业总量偏小，现代服务业发展缓慢。这既有基础条件差、工业化加速推进等客观因素，也有重视不够、推动不力等主观原因。可持续发展能力不强。重大投资项目偏少，部分项目推进缓慢；经济发展方式粗放，环境保护压力大，节能减排任务重；自主创新和成果就地转化能力弱，研发投入仅占生产总值的0.74%，低于全国平均水平；国有企业改革改制还需进一步深化，一些深层次矛盾需认真研究解决。一些涉及人民群众切身利益的问题还比较突出。劳动就业、就学就医、土地征用、城市拆迁、失地农民生活保障等方面还有不少问题亟待解决。发展环境还不够宽松。个别工作人员法制意识淡薄，服务意识不强，工作效率低下，不作为、乱作为现象时有发生；政府职能转变、作风建设有待进一步深化。这些问题必须高度重视，采取有效措施认真加以解决。

　　二、2008年工作总体要求

　　2008年是深入贯彻党的十七大精神的开局之年，也是全面实施“十一五”规划的关键一年。经济全球化和国际、国内产业转移加快，国家实施中部崛起战略、洛阳比照东北老工业基地享受优惠政策等，为我们加快发展创造了有利条件。经过多年积累，我市经济实力不断增强，城市形象显著提升，为今后发展奠定了坚实基础。同时，国家实施稳健的财政政策和从紧的货币政策，宏观调控力度加大，区域竞争日趋激烈，给我们带来了巨大压力。我们必须居安思危，未雨绸缪，抢抓机遇，趋利避害，努力开创经济社会又好又快发展的新局面。

　　2008年政府工作的总体要求是：认真贯彻党的十七大、中央经济工作会议和省委八届五次全会精神，全面落实科学发展观，大力实施工业强市、旅游强市、科教强市和项目引资双带动战略，转变经济发展方式，强力推进结构调整，继续扩大改革开放，高度重视改善民生，努力优化发展环境，加快工业化、城镇化和农业现代化进程，加快生态文明建设，促进经济社会又好又快发展。

　　全市经济社会发展预期目标是：生产总值增长13%；财政一般预算收入增长15%；全社会固定资产投资增长20%以上；社会消费品零售总额增长15%；外贸出口增长15%，实际利用外资增长20%，利用市外境内资金180亿元；城镇居民人均可支配收入增长8%，农民人均纯收入增长6.5%；单位生产总值能耗下降5.1%；新增城镇就业10万人，城镇登记失业率控制在4.5%以内；人口自然增长率控制在6.5‰以内。

　　上述目标是根据我市经济社会发展情况，经过认真测算确定的，在实际工作中不仅要确保完成，而且还要完成得更好。

　　做好明年工作，必须把握以下重点：一是着力推进结构调整和产业优化升级。按照农业优质高效、工业优化升级、服务业优先发展的思路，加快调整产业结构，逐步提高第三产业比重。农业方面要大力发展特色农业、高效农业、品牌农业，提高产业化水平，构筑现代农业体系。工业方面要发展壮大六大优势产业，积极培育高新技术产业、高成长性关联产业和特色产业，加快建设先进制造业基地和新型工业城市。服务业方面要完善加快服务业特别是现代服务业发展的政策措施，进一步扩大规模、优化结构、增强功能、提高质量。二是着力推进改革开放。坚持用改革的思路和办法解决发展中的矛盾和问题，加快重要领域和关键环节改革步伐，全面提高开放水平，努力构建充满活力、富有效率的体制机制。三是着力加快城镇化进程。按照完善功能、突出特色、提升形象的思路，在城市建设中更多地注入历史文化元素，加快基础设施建设，不断增强综合承载力和辐射带动力；以农业产业化为基础、工业化为主导、特色产业为支撑、园区建设为载体，不断壮大县域经济，加速城乡一体化进程。四是着力建设生态文明。加强资源节约和环境保护，统筹经济社会发展与生态城市建设，努力营造最佳人居环境、最佳发展环境、最佳旅游环境和最佳创业环境。五是着力构建和谐社会。拿出更多精力，投入更多资金，办好关系人民群众切身利益的实事，努力使全体人民学有所教、劳有所得、病有所医、老有所养、住有所居，逐步实现人人享有基本生活保障、人人享有基本医疗卫生服务的目标。

　　三、加快经济结构优化升级

　　坚持好字优先，转变发展方式，努力在优化结构、产业升级、提高效益、自主创新、协调发展等方面实现新突破。

　　(一)突出项目建设，增强发展后劲。坚定不移地实施项目引资双带动战略，全年计划实施重点项目954个，总投资2198.1亿元，完成投资725.9亿元。加快推进在建项目，积极协调解决项目建设中存在的问题，确保一批重大项目如期竣工投产。加快项目前期工作，加强与上级部门的沟通、协调，及早完善土地报批、环境评价、安全评价、消防评价等各项手续，争取项目尽快开工。立足比较优势和产业基础，积极谋划一批投资规模大、产业链条长、事关长远发展的高成长性关联项目，确保项目有序接替。坚持项目例会、领导分包项目等制度，多方筹措建设资金，加大项目协调、服务力度，为项目建设创造良好环境。

　　(二)调整产业结构，提升工业发展水平。实施千万元以上工业结构调整项目335个，总投资1025亿元，完成投资375亿元。加快发展六大优势产业。先进制造业重点抓好一拖工业园、中信重机特大型选矿设备产业化、LYC风力发电轴承等在建项目；开工建设中信新重机工程三期、北玻低辐射镀膜玻璃设备、河柴重工新能源气体发动机、轴研所精密重机轴承等项目，确保中色科技重大技术装备扩建、中信重机冶铸基地、南车集团大型铁路养护机械等项目竣工投用。铝精深加工产业重点抓好伊电集团50万吨连铸连轧、中铝公司20万吨热连轧等在建项目，开工建设新电集团40万吨铝板带箔等项目。能源产业重点抓好华阳孟津电厂2×60万机组、偃师嵩山及焦村各60万吨煤矿、洛宁和嵩县秸秆发电等项目，争取新电集团、豫能阳光二期2×30万机组热电联产项目尽快获国家核准；积极推进伊电集团压小上大2×60万机组、永骏新能源100万吨二甲醚等项目前期工作；确保义煤孟津、义安、新义各120万吨煤矿、黄河同力纯低温余热发电、禹门河水电站等项目竣工。石化产业重点抓好洛阳石化1000万吨炼油扩能等项目，争取45万吨PX、60万吨PTA项目获国家核准，积极做好20万吨聚丙烯等项目前期工作，加快推进石化工业基地建设。硅光电产业重点抓好中硅高科2000吨多晶硅、阿特斯3000吨长晶及硅片、613所光电研发基地、上海超日一期50兆瓦太阳能电池片、昇扬硅业20万吨工业硅等项目；开工建设中航光电连接器产业化基地、5715光电基地、中硅高科新2000吨多晶硅等项目。钼钨钛产业重点抓好洛钼集团4万吨钼精深加工等在建项目，开工建设725所2万吨钛板带等项目。着力培育高成长性关联产业和特色产业。重点发展太阳能、光电、生物制药等产业集群，抓好炘源晶太阳能、洛玻太阳能玻璃、普莱柯生物新疫苗产业化、伊龙药业1000吨生物制品等项目。加快发展汽车、拖拉机、摩托车、电力机车、飞机零部件和钢制办公家具、纺织、陶瓷、黄金等特色产业集群。加强经济运行协调。认真分析宏观经济走势和市场变化，做好重点企业、重点行业的跟踪监控。加强煤电油气运和资金等生产要素的供需衔接。狠抓企业扭亏增盈，最大程度地降低消耗、提高效益。重点抓好洛阳石化、中铝公司、洛玻、大唐洛热等大户的扭亏减亏工作。

　　(三)加快新农村建设，繁荣农村经济。加大涉农投入。全年市财政安排支农专款7700万元，比2007年增加945万元。认真落实支农惠农政策，加大扶贫开发力度，切实减轻农民负担，千方百计增加农民收入。加快发展现代农业。优质粮食面积达到350万亩，粮食总产稳定在200万吨左右；大力发展工厂化畜牧养殖业，搞好重大疫病防治，畜牧业占农业总产值的比重达到35%。扩大烟叶、中药材、水果、牡丹种植面积。叫响崤山烟叶、上戈苹果等一批品牌产品。大力发展绿色农业，建设一批无公害农产品基地和农业观光园。全面推行农产品质量安全市场准入。发展涉农龙头企业20家、农村专业合作组织50家。抓好循环农业示范市建设。加快农村基础设施建设。积极推进前坪水库前期工作，抓好中小型水库除险加固，新增有效灌溉面积3万亩、节水灌溉3万亩，治理水土流失200平方公里。深入开展“村村整治、千村达标、百村示范”工程和文明村、卫生村创建活动，建好200个示范村和300个重点整治村。根据村庄自然条件和经济发展状况，因地制宜，分类指导，加快农村饮水、道路、沼气、电力、文化、卫生等基础设施建设，通过典型引路，逐步推动有条件的村庄发展成为农村新社区，推动社会主义新农村建设取得突破性进展。

　　(四)突出旅游业，发展壮大服务业。发挥旅游业带动作用。精心办好第26届牡丹花会、第5届河洛文化旅游节、中国旅游交易会洛阳分会场。认真筹备2009年世界邮展。重点抓好龙门、隋唐城、汉魏故城等文化旅游产业园和伏牛山生态旅游区、小浪底-西霞院旅游度假区等建设，新增3A级以上景区5家。加快旅游资源整合，深化景区体制改革，吸引更多资本投资旅游业，形成文化旅游核心区和产业集群，打造区域性旅游中心城市和国际最佳旅游目的地。全年接待游客4245万人次，其中入境游30万人次；旅游总收入206亿元。大力发展商贸流通业。重点抓好申泰新世纪广场、亚威卓越国际广场等项目建设，新增商业面积30万平方米。加快中储和一拖物流园区、一运小浪底码头等项目建设。抓好“万村千乡”市场工程，扩大农村消费。着力培育现代服务业。加快发展金融、保险、证券、通讯、计算机服务、中介服务、法律咨询等新兴业态，不断提高现代服务业比重。积极发展生产性服务业。围绕现代工业、现代农业，大力发展产品设计、研究开发、市场营销、仓储运输等生产性服务业，形成工业、农业、服务业联动发展、相互促进的格局。稳步发展房地产业。深化住房制度改革，规范房地产市场秩序，完成房地产开发70亿元以上，开工商品房1000万平方米、竣工300万平方米。加快发展新区服务业。落实优惠政策，鼓励商贸流通、文化体育、娱乐休闲、金融保险、邮政通讯、公共交通、医疗卫生等服务业向新区集聚。积极引进便民超市、农贸市场、社区服务中心等服务项目，构建便民利民的服务体系。

　　(五)推进自主创新，提高核心竞争力。全年市财政安排科技专项经费2810万元，比2007年增加346万元。加强创新能力建设。制定优惠政策，鼓励企业加大研发投入，加强科研院所与大中型企业产学研联合，加快科研成果就地转化，形成具有自主知识产权的核心技术。新建全国、省、市级研发机构30个，进入省百户重点企业、50户高成长性企业和市30户重点企业要全部建立研发机构，争取一批企业列为国家、省创新型试点。支持中信重机、河科大联合申报国家级重点实验室。加快关键技术和重点领域科技攻关。实施科技攻关项目100个、关键技术60项，专利申请突破1600件；培育高新技术产品60项，高新技术企业40家，力争高技术产业占规模以上工业增加值的比重达到省平均水平。加快创新服务体系建设，优化创新环境，建立健全专项风险投资基金及退出机制，不断完善服务功能。加强知识产权保护。加快产业高地建设。做好伊洛工业园发展规划，拓展新的发展空间。全年高新区完成技工贸总收入630亿元，工业总产值550亿元。

　　(六)搞好节能减排，提高发展质量。认真落实节能降耗目标责任制。建立完善科学的节能统计体系，将节能减排指标分解到县(市、区)和企业，抓好重点行业、重点企业和重点领域的节能减排。完成20家重点排污企业污染源在线监测装置建设。严格执行项目环境准入制，科学制定能耗标准，严把项目审核关。综合运用产业政策、环境容量、差别电价等措施，推广节能环保新技术、新工艺、新设备，淘汰落后生产能力。做好机关办公楼和大型公共建筑节能工作，加快推进建材节能与小区节能改造。加强资源节约和综合利用。加快工业园区建设，推广多层标准化厂房，建成标准化厂房38万平方米，提高单位面积投资强度和产出效益。切实保护耕地，完成土地“三项整治”2万亩。深化矿产资源整合，基本完成小煤矿生产系统改造、省骨干煤矿与地方国有煤矿的整合。优化重点矿山资源配置，规范开采秩序。大力发展循环经济。全面推行清洁生产审核，推广循环先进技术，加强废旧资源再利用，工业固体废物综合利用率达到60%以上。启动煤炭行业循环经济推进计划，抓好示范企业、园区循环经济试点。

　　(七)发展县域经济，增强综合实力。立足比较优势，坚持工业兴县，加快培育骨干财源企业、特色产业和优势产业，不断提高县域经济占全市经济的比重和工业占县域经济的比重。支持县(市、区)加快工业园区、标准化厂房和重大项目建设，引导特色产业、民营企业向园区集中，实现集约发展。加快贷款担保体系建设，拓宽县域企业融资渠道。出台优惠政策，鼓励各县(市、区)在区域竞争中加快发展，晋位争先。

　　四、加快改革开放步伐

　　以国企改革为重点、以招商引资为抓手、以非公有制经济为突破，拓展对外开放的广度和深度，不断提高开放型经济水平。

　　(一)加快改革步伐，增强发展动力。抓好国有企业战略重组，通过增资扩股、股权转让等形式引进国内外战略投资者，实现投资主体多元化。重点做好洛铜、黄河水泥、中昊化肥、永安特钢等企业重组后的后续工作，积极推进一拖集团战略重组，加快实施洛轴集团和春都集团政策性破产。加快国有大中型企业辅业改制和分离企业办社会，全面完成企业中小学分离任务。继续推进国有中小企业改革改制，搞好资产处置和职工安置，妥善解决遗留问题。深化国有资产管理体制改革，确保国有资产保值增值。同时，加快农村义务教育、城市公用事业、水利、干线公路和县乡公路养护等管理体制改革，推进集体林权制度、土地流转制度改革。

　　(二)狠抓招商引资，提高开放水平。创新招商方式，建立职业化的招商引资队伍，积极开展驻地招商、上门招商和以商招商，不断提高招商水平。建立科学的业绩考核体系和经费保障机制，调动各级招商引资的积极性。拓宽招商领域，紧盯国内外500强和知名企业搞好推介，在重视工业项目招商的同时，加大基础设施和文化、旅游、商贸等服务业项目的招商力度，严禁引进高污染、低水平项目，实现招商引资向招商选资转变。注重招商实效，突出抓好牡丹花会、中博会、豫商大会、中国国际投洽会、香港豫洽会等招商工作，搞好项目后续服务，不断提高项目履约率和资金到位率。

　　加快发展开放型经济。优化出口商品结构，扩大电子、机电、矿山、冶金等高新技术产品和成套设备出口，扩大铝、钼、人造刚玉等资源性深加工产品出口，培育一批新型出口企业。引进国外先进装备、技术和关键零部件，促进产业升级。

　　(三)发展非公有制经济，增强发展实力。毫不动摇地鼓励、支持、引导非公有制经济发展，在放宽市场准入、改进金融服务、促进技术创新、健全服务体系、加强人才培养等方面，加大扶持力度。鼓励非公有制企业参与国有资本调整和战略重组。加强中小企业信用担保体系建设，抓紧组建创业投资公司，为民营企业搭建融资平台。力争非公有制经济增加值增长16%，营业收入超亿元企业达到110家，其中超5亿元企业20家，超10亿元企业6家。

　　(四)抓好财税金融，增强支撑能力。积极研究宏观政策，争取资金支持。充分发挥市场机制作用，努力搭建融资平台，筹措更多发展资金。理顺市县财政体制，大力培植财源，完善非税收入管理机制，优化收入结构，确保应收尽收。调整支出结构，加大民生投入，确保法定支出和社会保障支出。深化财政改革，加快建立公共财政体系，推进公共服务均等化。

　　发挥金融支持地方发展的作用，加强银企合作，引导各金融机构加大对城市建设、工业项目建设和新农村建设的支持力度，全年新增贷款90亿元以上。积极吸引域外金融、保险、证券机构在洛设立分支机构，争取引进1-2家股份制银行。加快筹建地方保险机构。健全农村金融服务体系，推进农村信用社改革，支持有条件的联社组建农村合作银行；开展农村金融机构试点，发展多种形式的信贷组织。搞好农业政策性保险试点和旅游景区保险试点。加强金融监管，优化信用环境，防范和化解金融风险。

　　加快企业上市步伐。重点支持中信重机、北玻公司完成上市，中硅高科、普莱柯生物、凯迈机电、亚洲啤酒、天源集团等省定重点后备企业加快上市步伐；支持市商业银行引进战略投资者，做好上市准备；抓住创业板推出后中小企业上市机遇，认真筛选上市后备企业，积极推进中小科技企业上市工作。

　　五、加快推进城镇化进程

　　加强规划指导，强化基础建设，完善城市功能，统筹城乡发展，努力实现城乡共同繁荣。

　　(一)科学编制规划，提升城市品位。突出城市特色，注重文化内涵，按照主体功能区规划要求，统筹城乡规划，继续做好第四期城市总体规划和土地利用总体规划修编，加强城市总体规划对县乡规划的指导，高标准做好区域控制性详细规划，不断扩大控制性详细规划的覆盖面。严格规划审批，切实维护规划权威。

　　(二)加快基础设施建设，完善城市功能。强力推进新区开发。全年完成投资100亿元以上，实施项目115个。重点开发开元大道两侧和开阳湖南侧组团，抓好中心区T形商业街、宝龙城市广场二期等项目。加快沿河百栋高层建设，确保累计开工135栋。高标准开发洛阳南站及周边区域，抓好关林商贸区组团升级改造。抓好太康路东延长线、瀛洲路污水处理厂及排污管网建设、瀛洲桥续建等工程，加快新区医院、新区高中和新区博物馆建设。力争完成洛宜铁路迁移改线工程。加快洛北城区改造。投资83亿元，实施128个项目。抓好亚洲城C区、中泰世纪花城三期、曼哈顿广场等续建项目和南昌路、长江西路交叉口地块等新建项目和城中村改造项目。出台补贴政策，引导各城市区加大道路建设力度，新开工、改扩建一批城市道路，重点抓好滨河路、瀛州北路、孙辛路、道北二路、衡山路和华山北路、九都西路打通等项目，积极谋划一批城市立交桥项目。加快涧西污水处理厂中水回用、垃圾处理及中转站等52个市政基础设施项目建设，同步配套各项基础设施，城市集中供热率达到25%，燃气普及率60%。

　　(三)加强城市管理，提高精细化水平。以创建全国文明城市为突破口，巩固创卫成果，深化城市管理体制改革，健全长效管理机制。加快建立数字化城市管理平台，完善市容巡查制度，集中开展市容市貌专项整治，创造整洁、优美、有序的城市环境。坚持公交优先，增加公交线路，规划建设停车场，加强交通管理，努力缓解交通拥堵现象。完善社区功能，启动市级社区服务信息网络体系建设，搞好物业五级管理，积极创建国家、省物业示范小区，建立规范、公开、透明的物业管理秩序，实现物业管理全覆盖。

　　(四)加快生态城市建设，打造最佳人居环境。继续实施碧水蓝天青山绿地工程，全面开展污染源普查，大力推进环保治理工程，确保化学需氧量、二氧化硫排放量分别削减2000吨和2万吨，全年城市环境空气质量二级以上天数300天。抓住林业生态省建设机遇，争取更多项目资金，强力推进林业生态市建设，加快环城和通道花卉苗木林果产业带、城郊森林建设。完善隋唐城遗址植物园二期、洛浦公园东延伸工程，抓好广场改造、街头绿地建设，新增绿地83公顷。完善新区景观水系。大力实施天然林保护、生态能源林等项目，完成营造林28万亩。科学规划，分期治理，加快生态洛河建设步伐。

　　(五)加快交通基础设施建设，提高通达能力。完成投资26亿元，重点抓好洛阳机场、连霍高速洛阳段改造，洛阳至吉利、偃师至洛宁快速通道及省道331改建等公路建设，建成洛阳至南阳高速汝阳段和宜阳、洛宁洛河大桥，改造省道323丁流至上天院段，建设县乡公路120公里。做好武西高速石人山至栾川、洛阳至栾川高速前期工作。加快推进农村客运网络建设。

　　(六)推进城乡一体化试点，加快农民进城步伐。完善以工促农、以城带乡机制，统筹城乡基础设施、公共服务、劳动就业和社会管理，缩小城乡差距，力争全市城镇化率达到42%。加快县城和小城镇基础设施建设，基本形成布局合理、各具特色、功能完善的县域城镇体系。制订优惠政策，加快城中村、城郊村改造步伐。出台就业、住房、医疗、养老等各项政策措施，鼓励城郊农民进城定居。搞好偃师、栾川、嵩县城乡一体化试点，吸引更多农民向县城和小城镇集中。

　　六、加快建设文化强市

　　大力发展文化产业，繁荣文化事业，建设和谐文化，塑造城市精神，不断增强城市文化软实力。

　　(一)挖掘城市文化内涵，彰显城市魅力。抓住国家实施大遗址保护机遇，积极申报洛阳片区大遗址保护项目，搞好隋唐城、汉魏故城保护规划，完成隋唐城定鼎门、汉魏故城阊阖门和偃师商城城墙遗址保护展示工程；启动隋唐城明堂、天堂及汉魏故城城墙保护展示工程。开工建设河南壁画馆和洛阳匾额博物馆。积极开展文物普查，加强历史文化街区、工业文化遗产保护。积极参与丝绸之路、大运河河南段申报世界文化遗产工作。

　　(二)大力发展文化产业，增强文化竞争力。编制文化产业发展规划，充分挖掘河洛文化、民俗文化、特色文化，整合文化资源，加快建设地域特色鲜明、产业优势明显的文化产业基地和产业集群。加快曲剧团综合演艺中心建设，创作一批有影响力的文化精品。积极发展广告艺术、文化创意、网络动漫等新型文化产业，支持发展娱乐休闲、艺术培训等社会文化产业。深化体育场馆管理体制改革，引导社会资金投入体育产业。大力发展体育彩票、健身俱乐部、体育中介等相关产业。加快发展广电产业，打造品牌栏目。

　　(三)深化文化体制改革，增强文化发展活力。认真落实配套政策，加快文化管理体制改革和经营性文化事业单位改革，引导文化团体走向市场。深化人事、分配制度改革，推行聘用制和劳动合同制，加快建立有利于调动文艺工作者积极性、创造性的管理体制和运行机制。多渠道培养高素质的文化经营管理人才。

　　(四)构建公共文化服务体系，丰富文化生活。深入开展广场文化狂欢月、全民读书月、送书下乡等群众文化活动，组织好“欢乐进农村、进社区、进军营”等公益性文艺演出。实施农村数字电影放映工程，全年放映公益性电影2.8万场；积极利用国家政策，加大基层文化设施投入，抓好23个综合乡镇文化站建设。

　　(五)建设社会主义核心价值体系，培育文明新风。积极创建文明城市、文明景区，扎实开展道德规范进万家、诚实守信万人行活动，建设诚信洛阳、礼仪洛阳、魅力洛阳。做好新时期双拥工作，落实优抚安置政策，充分发挥部队、民兵预备役在促进地方发展中的重要作用。大力弘扬厚重、包容、文明、开放、创新的城市精神，使之成为全市人民的自觉实践。

　　七、加快建设和谐洛阳

　　更加关注民生民情，更加注重和谐社会建设，切实解决好人民群众最关心、最直接、最现实的利益问题。

　　(一)全力办好市委、市政府承诺的20件实事，让人民群众得到更多实惠。为农民办的十件实事：一是搞好烟叶、奶牛等农业政策性保险试点，继续实行对农民的种粮直补和综合直补政策。二是改造农村困难群众危房1.4万间以上。三是建设通组通户道路5000公里、通村公路306公里、通村桥梁34座1934延米，解决113个行政村的联网道路；完善农村道路安保工程100公里。四是完成农村劳动力培训20万人次，转移130万人次；扶贫搬迁9000人。五是新建农村户用沼气10万座，实现沼气服务网点全覆盖。六是解决农村20万人饮水安全问题。七是新增乡级便民服务中心50个、农村社区服务中心200个，完成村镇规划500个。八是增加农村高学历教师比重，免除农村义务教育阶段学生学杂费和教科书费，为贫困寄宿生补助生活费，解决部分农村学校饮用水困难。九是改造建设标准化村卫生所150个；进一步完善新型农村合作医疗制度，参合农民435万人以上、参合率92%；免费为城乡群众做白内障手术1万例。十是新增农村低保对象1.5万人；低保补助标准不低于30元；新建、改扩建敬老院42所，五保对象集中供养率达到40%。为城镇居民办的十件实事：一是投资500万元，实施“菜篮子”工程。二是大力发展廉租住房和经济适用房，着力解决最低收入家庭住房问题。三是完成棚户区改造25万平方米、国有大企业住房改造12万平方米。四是改造背街小巷与城中村道路166条；开工建设孙辛桥，完成积翠路、启明南路、滨河北路南昌路至王城大道段工程；建成新区第一公交停车场。五是投资43亿元，加大城中村和旧城改造力度；建成城东区临时热源地，启动南关热源厂项目前期工作。六是完成城市区12所学校标准化建设，免除城市义务教育阶段学生学杂费，为城市义务教育阶段贫困学生免费提供教科书。七是实施涧河污染治理工程，解决市区河渠污染问题。八是新增城镇就业10万人、再就业4万人，保障困难群体就业1.5万人。九是对60岁以上老年人乘公交车实行优惠。十是实施民警驻勤制，强化社区平安建设。

　　(二)加快发展社会事业，促进经济社会协调发展。坚持政府主导，加大教育投入，全年市财政安排教育专项经费5.67亿元，比2007年增加9907万元。巩固“两基”成果，小学适龄人口入学率达到99%，初中入学率达到98.5%。深化教育改革，不断提高办学水平。加强新区学校、农村学校和薄弱学校建设。积极开展双语教学试点。认真解决城镇中小学班额过大问题。抓好边远山区农村中小学寄宿制试点。完善农村义务教育经费保障机制，保障困难家庭、进城务工人员子女平等接受义务教育。加快普及高中阶段教育，初中升入高中阶段比例达到72%。大力发展职业教育，不断提高办学层次，努力培养更多实用人才。积极支持高等教育发展。落实公共卫生补助政策，重点支持公共卫生、农村卫生和城市社区卫生，抓好县级医院建设，重点解决好贫困人口看病难问题。坚持医疗卫生公益性质，深化管理体制改革。加强食品药品监管，全面推行食品卫生监督量化分级管理，确保食品药品安全。推进全民健身计划，提高体育竞技水平；搞好2008年北京奥运会火炬传递活动，扩大洛阳影响，展示洛阳形象。实施村村通广播电视工程，启动县(市)有线电视数字化整体转换工作，扩大市、县级广播电视有效覆盖面。稳定低生育水平，提高人口素质，发展老龄事业。依法保障妇女儿童、残疾人和未成年人的合法权益。进一步完善困难群体司法救助制度。加强统计、审计、人防、气象、地震、地方志、民族宗教、外事侨务、新闻出版等工作。

　　(三)全面加强社会保障，切实解决好民生问题。全年市财政安排社会保障和就业资金1.29亿元，比2007年增加1634万元。抓好“菜篮子工程”。坚持一把手负责制，积极发展蔬菜种植和畜牧养殖，畅通农产品进城绿色通道，保障蔬菜、肉蛋奶等副食品供给；加强质量监管，让群众吃上放心菜；加强价格监管，依法打击各种价格违法违规行为；充分运用价格调节基金，完善和落实对低收入群众的补助办法，认真解决好影响民生的价格问题。千方百计扩大就业。落实就业再就业优惠政策，加强职业技能培训，完善创业服务体系，以创业促就业。重点解决好失地农民等困难群体、大中专毕业生和军转人员的就业问题。完善社会保障体系。做好企业养老保险征缴、扩面、清欠工作，确保参保总数达到70万人。加快建立职工工资正常增长和支付保障机制。深化事业单位养老保险改革，积极探索建立农村养老保险、城市失地农民养老保险制度。进一步扩大城镇居民基本医疗保险覆盖面。加快建立覆盖城乡的社会救助体系，发展社会福利事业和慈善事业。

　　(四)加强平安建设，确保社会大局稳定。完善平安建设工作体系、社会治安防控体系、严打整治体系和矛盾纠纷排查调处体系，加强社会治安综合治理。狠抓公安基础工作，完成公安“三基”工程建设任务，依法防范和打击违法犯罪活动，争创全国同类城市中最安全的城市。健全信访工作长效机制，进一步畅通诉求渠道，解决好人民群众关心的热点、难点问题。落实安全生产责任制，健全重大危险源和重大隐患排查机制，开展重点领域专项整治，确保安全生产形势平稳。加强应急管理，增强应对和处置突发公共事件的能力。

　　八、加强政府自身建设

　　加快制度创新和管理创新，不断优化发展环境，努力建设法治政府、效能政府、诚信政府、廉洁政府、服务政府。

　　(一)严格依法行政，规范行政行为。坚持依法治市，加强政府立法，全年提请市人大常委会法规草案3件，颁布政府规章5件。全面落实规范性文件审查备案制度。完善行政执法责任制，规范行政行为，强化执法监督，搞好行政复议。自觉接受人大及其常委会的法律监督、工作监督和政协的民主监督，严格执行人大及其常委会的决议、决定，认真办理人大代表议案、建议和政协委员提案，积极听取各民主党派、工商联、无党派人士和人民团体的意见，促进依法决策、科学决策、民主决策。加强基层民主建设，做好第六届村民委员会换届工作。

　　(二)创新管理体制，提高行政效能。转变政府职能，强化社会管理和公共服务，加强行政服务中心建设。深化审批制度改革，加快政府网站和电子政务建设，推行网上申报、网上预审、并联审批，扩大网上行政服务范围，缩短基层、企业与政府打交道时间。全面推进政务公开，建立健全重大事项社会公示、听证、质询制度，使政府决策更好地体现民意。

　　(三)加强诚信建设，树立良好形象。加快政府信用体系建设，建立失信惩戒机制、责任追究机制和赔偿机制。对省、市确定为群众办的实事，要明确责任，按期完成。认真落实对企业和外来投资者的优惠政策，全面兑现对人民群众的各项承诺。

　　(四)坚持从严治政，加强廉政建设。完善绩效考核，加强效能监察，确保政令畅通。落实党政机关工作人员行政过错责任追究办法，强化行政问责，严肃查处有令不行、有禁不止、作风不实等问题。深入开展廉政建设和反腐败斗争，进一步完善预防和惩治腐败体系，从源头上预防腐败。认真开展商业贿赂专项治理，严肃查处违法违纪案件。

　　(五)强化服务意识，优化发展环境。不断完善10项优化发展环境的长效机制，坚持无缺位工作制、双休日正常办理请办事项等制度，保证基层请办事项有报必理、有理必果、有果必复。坚持首席服务官、企业直通车、行政执法绩效考核制度，深入开展民主评议活动，不断优化政务环境。清理和规范各种收费、检查、评比和表彰活动，及时受理企业投诉，严查各种破坏发展环境的案件，倾力打造负担最轻、秩序最好、办事最快、信誉最高、群众和企业最满意的发展环境。

　　各位代表，新的一年里，让我们以十七大精神为指引，高举中国特色社会主义伟大旗帜，坚定信心，乘势而上，与时俱进，开拓创新，使我市各项工作跃上新台阶，实现经济社会又好又快发展！
